# First-trimester nutrition insights from the United Arab Emirate Birth Cohort Study (UAE-BCS): assessment of dietary intake, micronutrient profiles, and folic acid supplementation in Emirati Women

**DOI:** 10.1017/jns.2025.11

**Published:** 2025-03-13

**Authors:** Sharon Mutare, Maysm Mohamad, Jack Feehan, Leila Cheikh Ismail, Habiba I. Ali, Lily Stojanovska, Howaida Khair, Abdullah Shehab, Raghib Ali, Nahla Hwalla, Samer Kharroubi, Andrew Hills, Michelle Fernandes, Salama Al Neyadi, Ayesha S. Al Dhaheri

**Affiliations:** 1 Department of Nutrition and Health, College of Medicine and Health Sciences, United Arab Emirates University, Al Ain, United Arab Emirates; 2 Institute for Health and Sport, Victoria University, Melbourne, VIC, Australia; 3 Department of Clinical Nutrition and Dietetics, College of Health Sciences, Research Institute of Medical and Health Sciences, University of Sharjah, Sharjah, United Arab Emirates; 4 Nuffield Department of Women’s and Reproductive Health, University of Oxford, Oxford, UK; 5 Department of Obstetrics and Gynecology, College of Medicine and Health Sciences, United Arab Emirates University, Al Ain, United Arab Emirates; 6 Department of Internal Medicine, Faculty of Medicine and Health Sciences, UAE University, Al-Ain, United Arab Emirates; 7 Public Health Research Centre, New York University, Abu Dhabi, United Arab Emirates; 8 Faculty of Agriculture and Food Sciences, American University of Beirut, Beirut, Lebanon; 9 School of Health Sciences, College of Health and Medicine, University of Tasmania, Launceston, TAS, Australia; 10 Department of Paediatrics, University of Oxford, Oxford, UK; 11 Nuffield Department of Women’s & Reproductive Health, John Radcliffe Hospital, University of Oxford, Oxford, UK; 12 MRC Lifecourse Epidemiology Centre and Human Development and Health Academic Unit, Faculty of Medicine, University of Southampton, Southampton, UK

**Keywords:** Dietary intake, Early pregnancy, First trimester, Folic acid adherence, Macronutrient intake, Maternal dietary habits, Micronutrient intake, Supplement use, United Arab Emirates, AMDR, acceptable macronutrient distribution ranges, BMI, body mass index, GDM, gestational diabetes mellitus, hbA1c, haemoglobin A1c (glycated haemoglobin, HPLC, high-performance liquid chromatography, ICP-OES, inductively coupled plasma-optical emission spectrometry, IDF, International Diabetes Federation, LBW, low birth weight, LGA, large for gestational age, MENA, Middle East and North Africa, NTDs, neural tube defects, RDA, recommended dietary allowance, SGA, small for gestational age, UAE, United Arab Emirates, UAE-BCS, United Arab Emirates Birth Cohort Study, UEAU, United Arab Emirates University, WHO, World Health Organization

## Abstract

Maternal health and nutrition in early pregnancy play a vital role in the growth and development of the foetus. During this time, macro and micronutrients contribute to nutritional programming, which helps form the foundations of the foetus’s life course health outcomes. This study aimed to investigate dietary habits, macro and micronutrient intake, micronutrient status, and folic acid supplement adherence among Emirati pregnant women in their first trimester. Data were collected according to the UAE-BCS study protocol, which was set up to investigate maternal nutrition, health, child growth, and developmental outcomes within the first 1000 days. Pregnant Emirati women with singleton pregnancies within their first trimester of pregnancy (between 8 and 12 weeks of gestation) were enrolled. The 24-hour food recall method was administered to collect dietary intake. The maternal mean average age was 29 years. Participants had high adherence to supplementation during pregnancy compared to preconception. The mean energy intake was 1345kcal, and 56% of participants consumed saturated fats above the acceptable macronutrient distribution ranges (AMDR), while 94% consumed below AMDR for total fibre. The consumption of micronutrients was below the recommended dietary allowance (RDA). Biochemical results show a high prevalence of low haemoglobin (74%) and deficiencies in vitamin D (39%) and vitamin E (96%). There is a need for research into dietary patterns and influences in pregnant women in the UAE. Furthermore, investigations of knowledge practices and attitudes towards supplementation and the factors contributing to folic acid supplement use are needed to inform government strategies and interventions.

Within the 280 days of pregnancy, the first trimester is one of the most sensitive periods in the development of the embryo and the foetus.^(1,2)^ During this period, critical structural and functional organs are used to exchange nutrients and waste material between the mother and foetus, sustaining the foetus’s growth and development through the next 28 weeks.^(1,2)^ This period is also a sensitive window during which nutritional programming of the foetus occurs, which has consequences on health, metabolic profiles, growth, and development throughout the life course.^(3–8)^

Women of childbearing age are recommended to take 400 micrograms/day of folic acid (vitamin B9) at least 2 to 3 months before conception to reduce the risk of congenital disorders, which account for an estimated 240,000 deaths in newborn infants globally.^(9–11)^ Within the first trimester, folic acid plays a crucial role in the production, proliferation and differentiation of erythrocytes, and evidence suggests it significantly reduces the risk of congenital disorders such as neural tube defects (NTDs), cleft lip and palate.^(12–14)^ A higher dosage of folic acid supplementation (4-5 milligrams) is recommended for at-risk women who have diabetes, women with high BMI (equal to or above 30kg/m^2^), women on folic antagonist drugs such as anti-epileptic drugs, and women who have a previous history of giving birth to infants with NTDs.^(9)^ Although there is a scarcity of research on folic acid supplementation within the UAE, a few studies mention the adherence and compliance to folic acid supplementation. A 2010 study reported that only 8% of pregnant women consumed folic acid supplementation before pregnancy, and 77% took the recommended intake during pregnancy.^(15)^ A 2013 study exploring knowledge of the importance and benefits of taking folic acid supplementation stated that only 42% of Emirati women knew the importance and benefits of taking folic acid before conception, only 11% thought it should be taken before pregnancy, while 51% did not know.^(16)^

Due to the crucial role of folic acid in erythropoiesis, its deficiency also contributes to anaemia in pregnancy, along with iron deficiency, which manifests as low haemoglobin levels.^(17)^ Moreover, the physiological changes during pregnancy include an increase of maternal blood by approximately 50%, which may influence anaemia.^(18,19)^ The World Health Organization (WHO) reported the global prevalence of anaemia is 30% and 37% in pregnant women of childbearing age and in pregnant women, respectively.^(20)^ Several types of anaemia are prevalent, particularly among Emiratis and the broader Middle Eastern region. These include iron deficiency anaemia, sickle cell anaemia, thalassaemia, and vitamin deficiency anaemia. Iron deficiency anaemia is the most common type in the UAE, with a prevalence of 24% in pregnant women and women of reproductive age.^(21)^ Sickle cell anaemia has a prevalence of less than 50 per 100,000 in the UAE, while in some Middle Eastern countries, rates can be as high as 250 per 100,000.^(22)^ Thalassaemia, an inherited genetic disorder which affects haemoglobin production, is highly prevalent due to consanguinity and endogamy,^(23)^ with a significant carrier frequency in the Emirati population.^(24)^ The prevalence of β-thalassaemia in the UAE is estimated to be around 17%.^(24)^ Although specific prevalence data for vitamin deficiency anaemia are not readily available, it remains a significant health concern in certain populations. Anaemia has implications on the overall perinatal experience of pregnancy, which include tiredness, sleeping difficulties, palpitations and breathing difficulties, as well as the risk of complications during pregnancy, such as preeclampsia and the foetal growth and outcomes (i.e., intrauterine growth restriction, preterm birth, small for gestational age (SGA)).^(17)^

Micronutrient status during pregnancy is also associated with preterm births, which are reported to account for 1 in 10 births globally and are the leading cause of under-5 child mortality.^(25,26)^ The vasodilation capabilities of vitamin E on vessels ensure adequate transportation of nutrients to the foetus via the placenta, while zinc and vitamin D help in the prevention of pregnancy complications such as premature membrane rupture and preeclampsia.^(27–32)^ Perinatal vitamin E levels are suggested to influence the oxidative stress in the mother and infant. In contrast, high maternal levels of vitamin B12 during pregnancy have recently been linked to a high risk of autism in infants. Low levels are associated with gestational diabetes mellitus (GDM), preterm birth, and low birth weight.^(33–36)^ Micronutrient deficiencies during pregnancy are significantly associated with an increased risk of preterm birth. WHO research indicates that zinc supplementation during pregnancy can reduce the frequency of preterm birth by 14% and improve overall maternal and foetal health.^(37)^ Vitamin D insufficiency contributes to preterm birth by affecting the regulation of inflammatory responses and muscle function, which are critical for maintaining pregnancy.^(38–40)^ Low serum vitamin D levels are linked to poor foetal growth and impaired infant skeletal development.^(41)^ Inadequate calcium intake can impair foetal bone development, resulting in poor bone formation and preterm birth, increasing the risk of skeletal abnormalities.^(42–44)^ Magnesium is crucial for muscle function, including the uterine muscles, and helps prevent preterm labour.^(45)^ Adequate magnesium intake during pregnancy is reported to reduce the risk of preterm birth, while lower serum magnesium levels are linked to preterm labour.^(45,46)^ While the benefits of folic acid supplementation are outlined above, a recent meta-analysis by Li et al. (2019) showed that higher maternal folate levels and folic acid supplementation are associated with a reduced risk of preterm birth.^(47)^ Furthermore, calcium, magnesium, zinc, and copper deficiencies are also associated with adverse perinatal health, foetal growth, and birth outcomes.^(35,48–50)^

Low maternal protein intake is associated with low birth weight (LBW) and structural changes to the foetus’s liver. In contrast, high fat intake affects adipocyte metabolism, foetal growth, and fat mass in offspring.^(51–55)^ Iron deficiency is linked to both preterm birth and low birth weight due to anaemia, leading to fatigue and lack of energy due to insufficient oxygen transport in the blood.^(56,57)^ At the same time, energy deficiency during pregnancy can also contribute to poor foetal growth and preterm birth.^(54)^ Maternal diet and lifestyle preconception are not only associated with prenatal health and birth outcome, but a recent study also suggests it significantly influences the mother’s postnatal health and lifestyle habits over the following years.^(58)^ Hence, optimal preconception diet and lifestyle habits, such as a healthy balance that includes recommended portions of fruit and vegetables, high fibre, low fat, and minimally processed foods, as well as achieving and maintaining a healthy weight, are recommended.^(59,60)^ Consequently, adequate nutrition is essential for the organogenesis and development of the foetus, for the establishment of adequate immune response and healthy metabolic programming of the infant.^(61)^

Differential dietary patterns exist between cultures and geographies; as such, previous studies have alluded to differential patterns between high-income, middle income and low-income countries, for example, the comparison of prepregnancy health and behaviours, supplementation, and nutritional preparedness for pregnancy in the United Kingdom population (high-income) and other population groups such as Malawi (low-income).^(62)^ Such studies provide an understanding of maternal and infant health; however, longitudinal studies with detailed prospective dietary assessments in Emirati women are needed before their nutritional profile and its impact on foetal and infant development is fully understood. Therefore, this study aimed to investigate dietary habits, macro and micronutrient intake, micronutrient status, folic acid supplement use and adherence among a sample of Emirati pregnant women in their first trimester.

## Methods

The UAE-BCS was set up to investigate and profile maternal nutrition, health, child growth, and developmental outcomes within the first 1000 days of life. The methods for this birth cohort study are described in detail in the study protocol.^(63)^ The study aimed to recruit pregnant Emirati women attending prenatal visits in Abu Dhabi and the Northern Emirates. Pregnant Emirati women with singleton pregnancy, within their first trimester of pregnancy (between 8-12 weeks of gestation), with no history of twin pregnancies or miscarriages, with absence of significant illness or chronic conditions, and who did not take any regular medications were eligible for the study. Recruitment commenced in January 2021 at Kanad Hospital, Al Ain, Abu Dhabi. Eligible women with confirmed viable pregnancies were recruited while attending their first-trimester nuchal translucency (NT) ultrasound examination visit between 12-13 weeks.

### Sampling method

**Fig. 1.**
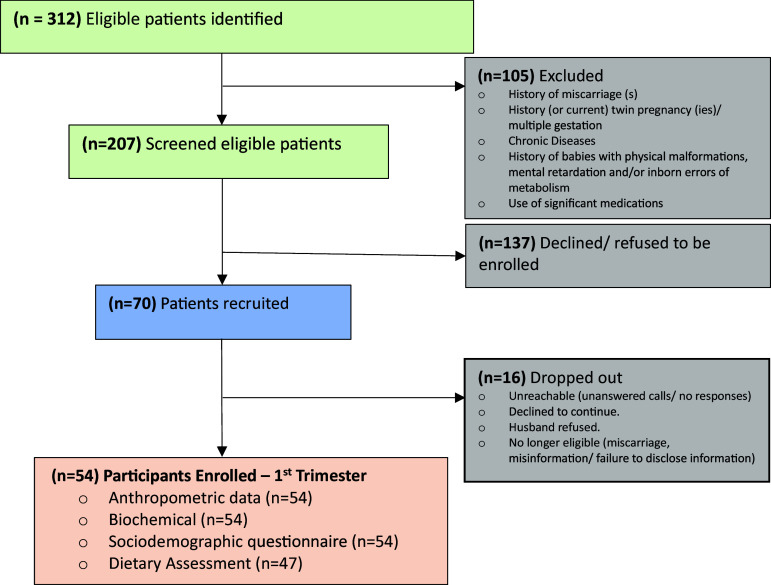
Flow diagram of eligible patients identified, screened, recruited, and enrolled into the study at 12-13 weeks gestation and the first trimester data collected and completed.

Overall, 312 women were identified, and 105 were excluded from the study at the recruitment stage due to not meeting the inclusion criteria. Of the 207 eligible women screened, 137 declined to participate at the enrolment stage. A total of 70 women were enrolled and agreed to participate in the study; however, 16 women were excluded for the following reasons: dropping out due to not wanting to continue data collection activities, husband’s refusal, lack of engagement (i.e. not answering calls or not showing up for hospital appointments), and becoming ineligible for the study (i.e. loss of current pregnancy, failure to disclose relevant information regarding eligibility at the recruitment stage such as previous twin pregnancies or previous miscarriages). The flow of the recruitment, enrolment inclusion and exclusion into the study are shown in Fig. 1; out of the 70 recruited patients, 54 were enrolled and participated in the study during the first trimester (12-13 weeks gestation).

### Data collection

The research team obtained anthropometric measurements, and blood samples were collected along with the first-trimester routine blood collection by the trained hospital phlebotomist. Only Emirati women who met the inclusion criteria described in the study protocol were enrolled, signed the consent and eligibility form, and provided sociodemographic data. The sociodemographic data included paternal and maternal information such as age, education level, accommodation type, and paternal health status.

Dietary data was collected on the day of the visit or within two days following the visit using the multi-pass 24-hour food recall method to collect food, beverages and supplements consumed within the last 24 hours.^(64)^ The ESHA Food Processor (v.11.0 Salem) was used to analyse dietary recall data into a nutrient composition which included energy (kcal), dietary fibre, macronutrients (carbohydrates, protein and fat) and micronutrients (iron, folate, vitamin B12, vitamin B1(thiamine), vitamin B2 (riboflavin), vitamin B3 (niacin), vitamin B6, biotin, choline, vitamin D, vitamin A, vitamin E, vitamin K, vitamin C, zinc, calcium, phosphorus, magnesium, copper, potassium, iodine, fluoride, chloride, selenium and manganese). The Institute of Medicine’s acceptable macronutrient distribution ranges (AMDR), recommended dietary allowance (RDA) and tolerable upper limits (UL) for adults were used to determine macronutrient and micronutrient distribution of the dietary data.^(65–70)^

Anthropometric measurements were obtained using a stadiometer (Seca, 213 portable stadiometer) for height and the Tanita (Tanita BC-418, Tanita Corp., Tokyo, Japan) scale for weight and BMI calculated as described in the study protocol. The WHO cut-off recommendations for BMI were used to classify weight within the study group: <18.5 is underweight, 18.50 – 24.99 is normal, ≥ 25 is overweight, and ≥ 30 is obese.^(71)^

Additionally, medical records were used to attain folic acid supplement use and type (multivitamin or folic acid only); time supplementation began (prior or during pregnancy); conception type, whether it was assisted (IVF) or spontaneous; parity; and consanguinity defined as first or second degree. For this study, prior supplementation is defined as use before conception, while during is defined as use after confirmation of pregnancy.

A trained hospital phlebotomist collected blood samples during the visit; samples were transported to the UAEU laboratory, where they were centrifuged (serum and plasma) and stored in a freezer at -80 until analysis. Biochemical analysis of blood samples for folate, ferritin, vitamin B12 and vitamin D was performed using the Cobas e411; the inductively coupled plasma-optical emission spectrometry (ICP-OES) was used to analyse lead, calcium, copper, magnesium, zinc, and iron; vitamin E was analysed using the high-performance liquid chromatography (HPLC); and the Cobas c111 was used to analyse whole blood for hbA1c levels. The cut-off used for the biochemical analysis, according to the American Board of Internal Medicine, laboratory test references ranges for a normal population.^(72)^

### Data analysis

Statistical analysis was performed using the SPSS software version 28.0 (SPSS, Chicago, IL, USA). The numerical variables are presented as mean and standard deviation when normally distributed, median and range (maximum, minimum) when skewed, and categorical data are expressed as counts and percentages. p-values < 0.05 were considered statistically significant. Cross tabulations and chi-square tests were used to determine the association between categorical variables related to the type and timing of supplement usage. Logistic regression was applied to explore the associations between nutrient deficiency and not meeting AMDR and RDA with sociodemographic characteristics (analysis is shown in the Supplementary file (Tables 5-12).

## Results

The maternal average age was 28.5 years; the mean average prepregnancy weight was 68 kg (Table 1). 41% had normal BMIs, 9.3% were underweight, while 17% and 33% were categorised as living with overweight and obesity, respectively. Around 40% of women had 2 or more living children, 28% had one child, and 32% were primigravida. Most pregnancies were spontaneous (93%). Almost half (48%) of participants were educated to high school and college diploma level, 44% had an undergraduate degree, and only 7% had postgraduate degrees. Most participants were unemployed or retired, while 21% were full-time, 2% were self-employed, and 7.5% were still students. For paternal education, 48% had high school and college diplomas, 48% had undergraduate bachelor’s degrees, and only 4% had postgraduate degrees. Furthermore, 89% of fathers were working full-time, 6% were either retired or unemployed, and 2% were students working part-time and self-employed, respectively. The consanguinity level for first or second and distant cousins was 43%.

**Table 1.** Baseline characteristics of the UAE-BCS. Demographic information (n=54)

Characteristics	Total *n* = 54	
	Mean	SD
Clinical characteristics		
Maternal age (years)	28.5	5.2
Height (cm)	158.6	5.7
Prepregnancy weight (kg)	68.2	19.8
BMI (kg/m^2^)	27.2	7.3
BMI <18·5 (kg/m^2^)	9% (*n* = 5)	
BMI 18·5 – <25 (kg/m^2^)	41% (*n* = 22)	
BMI 25 – <30 (kg/m^2^)	17% (*n* = 9)	
BMI ≥30 (kg/m^2^)	33% (*n* = 18)	
Parity		
First pregnancy	32% (*n* = 17)	
1 living child	28% (*n* = 15)	
≥2 living children	41% (*n* = 22)	
Conception type		
Assisted (IVF)	7 % (*n* = 4)	
Spontaneous	93% (*n* = 50)	
Maternal Education level		
High school/ College & Diploma	48 % (*n* = 26)	
Bachelor University Degree	44% (*n* = 24)	
Postgraduate (Master’s or Ph.D.)	7% (*n* = 4)	
Maternal Employment Status		
Student	7 % (*n* = 4)	
Full-time employment	21% (*n* =10)	
Self-employed	2% (*n* = 1)	
Unemployed or retired	70% (*n*=38)	
Paternal Education Level		
High school/ College & Diploma	48% (*n* = 26)	
Bachelor University Degree	48% (*n* = 26)	
Postgraduate (Master’s or Ph.D.)	4% (*n* = 2)	
Paternal Employment Status		
Student	2% (*n* = 1)	
Part-time	2% (*n* = 1)	
Full-time employment	89% (*n* = 47)	
Self-employed	2 % (*n* =1)	
Unemployed or retired	6% (*n* = 3)	
Consanguinity (Yes)	43% (*n* = 23)	

Table 1 shows maternal and paternal demographic characteristics with values presented as mean, standard deviation (SD) or absolute and relative frequencies and percentages with frequencies.

**Fig. 2.**
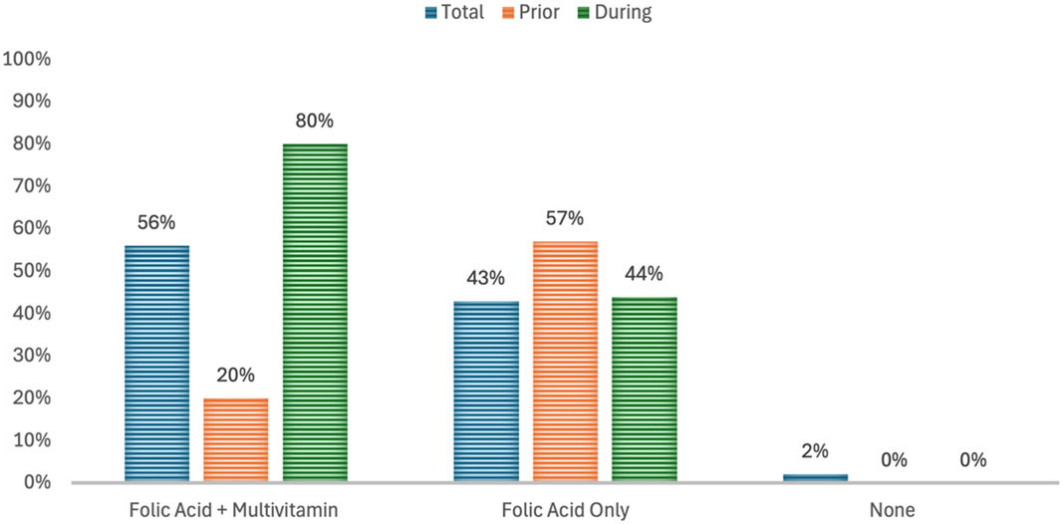
Supplement use and timing among participants. Prior to conception is defined as before conception, during is defined as after confirmation of pregnancy from a healthcare provider, and none is defined as no supplementation at all. Folic acid + multivitamin refers to the intake of a supplement multivitamin containing folic acid, and folic acid only refers to a supplement containing only folic acid.

As shown in Fig. 2, only 2% of the participants did not take any supplementation before or during pregnancy; 98% of participants took supplementation (57%) before and (44%) during pregnancy; 56% took a multivitamin containing folic acid, and 43% took a folic acid-only supplement. Of the women who took folic acid supplements, 57% started before the current pregnancy, and 44% started taking folic acid during pregnancy. At the same time, 80% of the participants who took supplementation in the form of multivitamins with folic acid started during pregnancy. Chi-square analysis showed a significant difference between supplement use and timing (p<0.001).

Table 2 shows that 65%, 90% and 60% of women were within the AMDR for carbohydrates, protein, and fats, respectively. Meanwhile, 17%, 10%, and 13% were below the AMDR for carbohydrates, protein, and fats. Only 19% exceeded their AMDR for carbohydrates and 28% for fats. For saturated fats, 56% were above the AMDR. Only 2% of women were within the daily recommendation for total dietary fibre.

**Table 2.** Maternal macronutrient intake table, the contribution of each macronutrient to energy (protein, carbohydrates, and fat), also shows the percentage of women below, within and above AMDR recommendations

Macronutrient	Mean	SD	AMDR	% of Women below the AMDR	% of Women within the AMDR	% of Women above the AMDR
Energy	1344.5	622.8	.	.	.	.
Carbohydrates	189.6	101.6	45 – 65 %	17	65	19
Protein	49.9	28.3	10 – 35 %	10.	90	0
Fats	43.7	22.0	20 – 35%	13	60	28
Saturated Fats	15.3	8.8	Less than 10%		44	56
Total fibre	9.9	10.1	28g/day (RDA)	94	2	4

As per IOM, macronutrient intake is shown as mean, standard deviation, and percentage of women consuming below, within, and above AMDR.

As displayed in Table 3, 100% of participants did not meet the recommended intake for iron, folate, biotin, choline, vitamin D, vitamin E, fluoride, and chloride. Moreover, the percentage of women who did not meet the RDA were calcium (98%), vitamin B2 and zinc (98%), vitamin K (98%), magnesium and vitamin A (96%), vitamin B12 (94%), potassium (89%), vitamin B6 (89%), vitamin B3 (87%), copper (87%), manganese (86%), vitamin B1 (83%), vitamin C (81%), phosphorus (78%), selenium (77%), and sodium (27%). While a few met their daily recommended intake, vitamin B3(11%), vitamin B6 (11%), vitamin A (4%), vitamin C (19%), zinc (2%), calcium (2%), phosphorus (22%), copper (13%), sodium (40%), iodine (6%), selenium (23%), and manganese (14%). Participants exceeded the tolerable upper intake level (UL) for some of the following micronutrients: vitamin B3 (2%), magnesium (4%), potassium (11%), sodium (33%) and iodine (3%).

**Table 3.** Micronutrient intake and adherence to IOM recommendations. Also shows the percentage of women below, within the recommended dietary allowance (RDA), and the percentage of women exceeding the tolerance upper intake level

Micronutrient	Mean	SD	RDA	UL	% of women below the RDA/ AI(*)	% of Women meeting the RDA/ AI(*)	% of Women exceeding the UL
Iron	8.3	4.8	27mg/day	45mg/day	100	0	0
Folate	112.9	125.9	600mcg/day	1000mcg/day	100	0	0
Vitamin B12	1.02	1.5	2.6mcg/day	N. D	94	7	**
Vitamin B1 (Thiamine)	0.7	0.8	1.4mg/day	N. D	83	17	**
Vitamin B2 (Riboflavin)	0.5	0.4	1.4mg/day	N. D	98	2	**
Vitamin B3 (Niacin)	8.4	8.2	18mg/day	35mg/day	87	11	2
Vitamin B6	0.7	0.7	1.9mg/day	100mg/day	89	11	0
Biotin	2.3	3.9	30mcg/day	N. D	100*	0*	**
Choline	86.1	83.1	450mg/day	3500mg/day	100*	0*	0
Vitamin D	1.2	1.7	10mcg/day	15mcg/day	100	0	0
Vitamin A	165.9	201.6	770mcg/day	3000mcg/day	96	4	0
Vitamin E	1.4	1.6	15mg/day	1000mg/day	100	0	0
Vitamin K	20.3	31.7	90mcg/day	NONE	98*	2*	**
Vitamin C	51.2	64.8	85mg/day	2000mg/day	81	19	0
Zinc	3.1	2.8	11mg/day	40mg/day	98	2	0
Calcium	348.7	234.7	1000mg/day	2500mg/day	98	2	0
Phosphorus	472.8	382.9	700mg/day	3500mg/day	78	22	0
Magnesium	103.8	112.1	350mg/day	350mg/day	96	0	4.3
Copper	442.7	512.7	1000mcg/day	10000mcg/day	87	13	0
Potassium	1059.1	1024.4	2900mg/day	NONE	89*	0*	10.6
Sodium	2372.2	2256.3	1500mg/day	2300mg/day CDRR*	27*	40*	33
Iodine	63.8	218.0	220mcg/day	1100mcg/day	91	6	3
Fluoride	0.24	0.29	3mg	10mg/day	100*	0*	0
Chloride	117.7	123.2	2300mg/day	3600mg/day	100*	0*	0
Selenium	44.2	63.0	60mcg/day	400mcg/day	77	23	0
Manganese	0.9	1.2	2.0mg/day	11mg/day	86	14*	0

Micronutrient intake is shown as mean and standard deviation and percentage of women consuming below, within the and above tolerable upper intake level (UL) as per the IOM recommendations.^(66–69)^ Adequate Intakes (AI) presented as *. No tolerable upper intake (UL) presented as **.

Our results show that approximately 74% of women had haemoglobin levels below the normal range of 12–16 g/dl, while only 26% were within the normal range (Table 4). For iron levels, 22% were within the normal range, and 78% were above the range.^(72)^ For folate, only 1.9% were below the range of 1.8 – 9.0ng/ml, 44% were within the range, and 54% were above.^(72)^ More women (96%) were below the range, and a few (4%) were within the range of 5.5 – 17.0 micrograms/mL for vitamin E, while most (96%) were within the range of 1.92-3.12 mg/dL for magnesium and only 4% were below the range.^(72)^ All participants (100%) were above the range for zinc (75 – 140 micrograms/dL), calcium (8.6 – 10.2mg/dL) and copper (100 – 200mcg/dL), and all were below for lead.^(72)^ Only 6% of women were above the hbA1c range of 4-5.6%, and 94% were within the normal ranges.^(72)^

**Table 4.** First-trimester micronutrient status of participants using ranges of normal population

Biochemical analysis	Mean	SD	1^st^ trimester - Cut-off ranges for the normal population	% of Women below the range	% of Women within the range	% of Women above the range
Haemoglobin(g/dL)	11.4	0.8	12 – 16 g/dl	74	26	0
Folate (ng/ml)	10.7	6.1	1.8 – 9.0 ng/ml	2	44	53.8
Ferritin(ng/ml)	61.3	56.6	24 – 307ng/ml	35	65	0
Vitamin B12 (pg/ml)	426.9	141.1	200 – 800pg/ml	2	96	2
Vitamin D(ng/ml)	32.7	9.3	30 – 60ng/ml	39	61	0
Vitamin E(mcg/ml)	2.7	1.7	5.5 – 17.0 micrograms/mL	96	4	0
Zinc(mcg/dl)	498.3	346.9	75 – 140 micrograms/dL	0	0	100
Lead(mcg/dL)	0.011	0.0	<5.0 micrograms/dL	100	0	0
Calcium (mg/dL)	16.7	2.0	8.6 – 10.2mg/dL	0	0	100
Copper (mcg/dL)	433.5	151.7	100 – 200mcg/dL	0	0	100
Iron (mcg/dL)	197.3	61.4	50 – 150mcg/dL	0	22	78
Magnesium (mg/dL)	2.5	0.3	1.92–3.12 mg/dL	4	96	0
hbA1c (%)	5.1	0.4	4–5.6%	0	94	6

The micronutrient status in participants was reported as mean and standard deviation, with cut-off points below, within, and above the range, as included according to the American Board of Internal Medicine, ABIM Laboratory Test Reference Ranges, July 2023.^(72)^

Logistic regression analysis of the dietary nutrient intake and biochemical biomarkers with baseline sociodemographic characteristics showed an association between hbA1c status and paternal education (OR=16.73; 95 % CI 1.14-245.36; p=0.04) and maternal education (OR=25.09; 95% CI 1.84-341.97; p=0.02). Moreover, there was a significant association between fluoride and paternal employment (p=0.01) and selenium with supplement type (OR = 0.09; 95 % CI 0.01-0.79; p=0.03) (multivitamin or folic acid only) (Supplementary Tables 6, 11 and 12)

## Discussion

In this study of 54 Emirati women in their first trimester of pregnancy, we report higher adherence to folic acid supplementation during pregnancy than before conception, high intake of saturated fats and high prevalence of women not meeting RDA for micronutrients. Additionally, deficiencies in vitamin D and E and low haemoglobin levels were observed in the study population.

In our study, 80% of participants took folic acid as a multivitamin, while 44% took a folic acid-only supplement during pregnancy. Compared to an earlier study in the UAE, our study shows more women currently adhere to folic acid supplementation during pregnancy than the 45% reported in 2003.^(73)^ A 2010 study from the UAE reported that 8% took folic acid prior to conception, while 65% took it after the first month of pregnancy; in comparison, our study shows an increase in supplementation prior to pregnancy and during than previously observed.^(15)^

The results reported in our study population are comparable to reports from other Arabic countries. Our results were comparable for folic acid supplementation during pregnancy (UAE-BCS 80%; Jordanian study 86%; Algerian study 83-94%).^(74–76)^

Our results show high adherence to supplementation compared to no supplementation prior to or during pregnancy (2%). We recommend implementing maternal nutrition and health campaigns and educational initiatives in the UAE to raise awareness about the benefits of folic acid supplementation. These efforts should particularly emphasise the importance of taking folic acid prior to conception, as it is crucial for the neural tube development of the foetus. Additionally, educating women about the significance of iron supplementation to prevent anaemia and support overall maternal and foetal health is important. Increasing knowledge about these vital nutrients will improve maternal and foetal health outcomes.

The pace of nutrition transition varies between regions and countries, with the UAE facing a rapid transition from traditional dietary and food habits and lifestyle practices to Western diets and more sedentary lifestyles.^(77)^ This transition presents public health challenges such as obesity and non-communicable diseases. The World Obesity Atlas (2023) has projected a rise in the prevalence of obesity in women to over 40% and 23% from 11% for adolescent children by 2035 in the Middle East and North African region.^(78)^ Maternal obesity is associated with childhood obesity in offspring.^(79)^ In our study, 50% were living with overweight and obesity according to the WHO classification.^(71)^ Prepregnancy BMI is associated with gestational weight gain and maternal obesity and influences complications during pregnancy, such as gestational diabetes, and adverse birth outcomes, such as macrosomia. Hence, raising awareness of a healthy lifestyle is vital to manage and maintain a healthy BMI before conception to enhance a positive pregnancy experience and outcomes.

To support foetal development and substantial recommended weight gain in women of normal BMI, they should consume additional calories in the latter trimesters and do consistent physical activity.^(80)^ However, within the first trimester, women should maintain adequate calorie intake to meet metabolic and physiological changes.^(65)^ However, our results show a lower average energy intake (1345kcal/day) than the daily recommended for non-pregnant women, which is much less than the intake of women in the same region.^(81)^ In this study, we examined the energy contribution of each macronutrient to energy. Tayyem et al. (2019) similarly examined first-trimester macronutrient energy contribution, and similar results are reported for protein, with none of the women consuming the above recommendation, only a few consuming below. Our study found a significant number of women consumed more saturated fats and low total dietary fibre, and saturated fats are associated with complications during pregnancy, foetal growth restriction and adverse birth outcomes such as LGA.^(82)^ These findings are supported by a similar study that suggests that women in the UAE either consume a diverse or Western dietary pattern, with the diverse pattern consisting of more fruit and vegetable intake as opposed to the Western that consists of fast foods and sweets.^(83)^ This is reflected in our study findings; over half (56%) consumed above the AMDR for saturated fats, while most (94%) did not meet the total fibre recommendation, suggesting women in our study consume a Western dietary pattern. Low dietary fibre intake during pregnancy in the United States of America study, a high-income western country, is observed in a study reporting similar findings to our study of lower dietary fibre intake of 17g/day than the recommended 28g/day, the results from our study show a much lower average intake of 10g/day.^(84)^

In contrast, a recent study in Ethiopia, a low-income country, observed low dietary fibre intake in pregnant women who consumed cereals and grains despite their high fibre content. This was attributed to the low number of women consuming fruit and vegetables within the study group and could be the contributing factor to our study observations.^(85)^ The consumption of high dietary fibre in Mediterranean diets is associated with a low risk of GDM in Iranian women.^(86)^ The adequacy and sources of dietary fibre in the UAE diet need to be investigated further.

The majority of women in our study were within the ADMR recommended intake for energy for protein, carbohydrates and fats, with only a below 10% for protein, 17% for carbohydrates and 13% for fats; some were above 19% for carbohydrates and 28% for fat, respectively. These are similar results to the Tayyem et al. study, which reported more women above recommendation for fat as seen in our study compared to none above for protein, a few above for carbohydrates and a small percentage below for both nutrients.^(81)^ However, it is important to note a significant limitation in our study’s methodology: we used a single 24-hour recall to collect dietary data. This approach may not accurately capture the usual intake and can lead to substantial variability in the results. Consequently, these findings are not generalisable. Future studies should employ more thorough and complementary dietary data collection methods, such as multiple 24-hour recalls or food frequency questionnaires, to improve the accuracy and reliability of dietary assessments.

The UAE is one of the countries undergoing an advanced nutrition transition in the Middle East, which, in addition to the implications of obesity, also faces moderate undernutrition and micronutrient deficiencies.^(77)^ Intakes below RDA for all investigated micronutrients were observed in this study apart from sodium, which 33% exceeded the UL. Similarly, a few studies observed similar results in micronutrient intakes lower than the recommended intakes in pregnant women, with one study from the region, in particular, reporting more women above the UL for sodium, the same as our findings.^(81,87,88)^ High sodium intake preconception is associated with hypertensive disorders in pregnancy and the outcome of SGA in infants.^(89)^ Adequate micronutrient intakes are required for foetal growth and development in early pregnancy, such as folate, iron and vitamin B12 in erythropoiesis^(90)^; vitamin D in foetal and maternal calcium homeostasis and organogenesis^(91,92)^; vitamin C in its role in preventing maternal pregnancy complications such as preeclampsia^(93)^; and an appropriate intake of vitamin A for the ocular and immunological health of the foetus.^(94)^ However, this population group’s dietary patterns and behaviours need to be investigated further, and their knowledge of micronutrient interactions can lead to poor bioavailability even with supplementation, such as iron interaction with vitamin C and calcium. Given the importance of micronutrients and their impact on fertility, maternal health, foetal growth and birth outcomes, it is essential to provide nutritional education and counselling and promote a diverse dietary pattern to women before and during pregnancy.

A recent review discloses folate, iron, and vitamin D as the most prevalent deficiencies in women of childbearing age and pregnant women in the Middle East.^(77)^ The highest micronutrient deficiency found in our study was vitamin E (94%), followed by vitamin D (39%), of which, for both nutrients, all participants did not meet the dietary requirements. Vitamin D is the most reported vitamin in literature due to the hypovitaminosis D situation observed in the Middle East.^(77,95)^ Our results show a low vitamin D intake in pregnant women despite reports from a recent study of female university students suggesting that a decent number of Emirati students consume milk, cheese, and vitamin D supplements.^(96)^ However, modern dietary changes that result in low dietary diversity may contribute to low sources of vitamins, including vitamin D. Vitamin D deficiency is reported as a common issue in the country in women, mainly due to the traditional clothing that covers the whole body and low engagement with outdoor activities, resulting in low exposure to sunlight ^(97)^ The outcomes of serum vitamin D in the UAE are attributed to a lack of knowledge of the sources of vitamin D to consume during pregnancy and low supplementation, in addition to spending less time outdoors and using sunscreen significant between trimesters for folate, ferritin, vitamin B12 and zinc.^(98)^

Our results also showed a high prevalence of low haemoglobin (74%); even though the majority of the participants are above or within the ranges for folate, vitamin B12 and iron, conditions such as thalassaemia need to be considered and investigated to determine a cause of anaemia.^(99)^ High ranges of calcium, copper, iron, folate and zinc can be attributed to supplementation with multivitamins, which our results show is high before and during pregnancy. Our results also suggest no or minimal lead exposure within the population group. Recent literature indicates that underreporting in dietary recalls may influence the results of research on nutrients and energy intake,^(100)^ and this underreporting may result from forgetfulness and inaccuracy in reporting portions, recipes, and weights of food. According to the International Diabetes Federation (IDF), 1 in 6 adults live with diabetes in the MENA region, 1 in 3 adults living with undiagnosed diabetes, and 1 in 7 live births are affected by hyperglycaemia during pregnancy.^(101)^ While our results show that only 6% of participants had hbA1c levels above the normal ranges, with 94% within the normal ranges, we found an association between hbA1c levels and maternal and paternal education levels. A similar result has been observed in a few studies, notably a 2023 Dubai study, which reports the level of education as a factor affecting hbA1c levels.^(102)^ The study observed high levels of HbA1c in illiterate individuals compared to those educated at the university level.

A 2019 study from India also reported that education significantly affects hbA1c levels independent of other factors such as income and background.^(103)^ Hence, recommendations for health education in health centres to improve awareness are appropriate for addressing the issue. Although high daily fluoride intake is associated with adverse pregnancy and birth outcomes and lower intelligence quotient (IQ) in children, adequate amounts of fluoride are beneficial for dental and bone health.^(104–106)^ In this study, fluoride intake was lower than the RDA for all participants, and the association was found with paternal education. In the UAE, fluoride levels in drinking water and other beverages were reported to be lower than recommended.^(107)^ Therefore, low fluoride intake can be attributed to consuming water and beverages with lower than recommended fluoride levels. At the same time, the association with paternal education can be attributed to knowledge of benefits leading to the purchasing and consuming beverages with low fluoride content. Selenium intake was lower than RDA in this study for most participants (77%).

Additionally, this study found an association between selenium and supplement type. Given that we investigated supplement type (multivitamin or folic acid only), the association with selenium may be due to the belief that taking multivitamins will provide needed nutrients, including selenium, instead of other dietary sources during pregnancy. Recent literature suggests that underreporting in dietary recalls may influence the research results on nutrients and energy intake.^(100)^ This underreporting may result from forgetfulness and inaccuracy in reporting portions, recipes, brands and weights of food. Given that lower than adequate nutrition in pregnancy has great implications on foetal development risk of congenital defects (NTDs and Cleft Lip), adverse birth outcomes (preterm, SGA and LBW), pregnancy complications (anaemia and preeclampsia), and long-term outcomes (childhood cognitive and behavioural outcomes) these deficiencies and dietary inadequacies need to be addressed to ensure maternal and infant health and a healthy population.

One of the main strengths of this study is that it is based on a population-based prospective observation setting, which helps to understand the characteristics of the Emirati population, particularly pregnant women. Although similar cohort studies exist in the UAE, to our knowledge, this is the first nationally representative study exploring maternal nutrition and status within the first trimester. All questionnaires used were predefined and validated, and trained researchers administered them. Researchers were trained to collect anthropometric and dietary data. Our findings identify research gaps which lead to the development of future projects that enhance maternal and infant health. There is a need for a larger sample and study group that includes different Emirates and applies proper sampling calculations and strategies. Another limitation is the use of one dietary assessment method. Although the 24-hour recall is a gold standard, it has its limitations; however, the multi-pass method was used to reduce bias.^(64)^

### Conclusion

The dietary patterns of first-trimester Emirati women in 2020, although improved from previous years, remain deficient in specific nutrients. These findings align with other Arab populations and underscore the need for (1) further research into the socio-cultural causes of these deficiencies, (2) educational initiatives to improve the nutritional health of first-trimester women, and (3) targeted prenatal supplementation campaigns.

In addition, we recommend public health measures aimed at women capable of becoming pregnant to increase the use of folic acid and iron supplements before conception. Ensuring adequate baseline levels of these nutrients, as well as vitamin D, and promoting appropriate weight gain during pregnancy, are essential for optimal maternal and foetal health outcomes

## Supporting information

Mutare et al. supplementary material Mutare et al. supplementary material

## Supplementary material

The supplementary material for this article can be found at https://doi.org/10.1017/jns.2025.11
