# Supplementary material for: First-trimester nutrition insights from the United Arab Emirate Birth Cohort Study (UAE-BCS): assessment of dietary intake, micronutrient profiles, and folic acid supplementation in Emirati Women
Source: J Nutr Sci. 2025 Mar 13;14:e24. doi: 10.1017/jns.2025.11 (PMC11950705; doi:10.1017/jns.2025.11)
Supplement: Mutare et al. supplementary material [file S2048679025000114sup001.docx]

| PARAMETER | BIOCHEMICAL BIOMARKER | | | | | | | | | | | | | |
| --- | --- | --- | --- | --- | --- | --- | --- | --- | --- | --- | --- | --- | --- | --- |
|  | Hemoglobin | | Folate | | Ferritin | | Vitamin B12 | | Vitamin D | | Vitamin E | | Zinc | |
|  | P. Value | Odds ratio (95 CI) | P. Value | Odds ratio (95 CI) | P. Value | Odds ratio (95 CI) | P. Value | Odds ratio (95 CI) | P. Value | Odds ratio (95 CI) | P. Value | Odds ratio (95 CI) | P. Value | Odds ratio (95 CI) |
| Maternal age | .33 | .72(.37-1.40) | .99 | .00(.00-.) | .49 | .83(.47-1.44) | .19 | .19(.02-2.25) | .05 | 19.2(.99-3.72) | .33 | 1.89(.53-6.79) | .92 | .97(.56-1.69) |
| BMI | .62 | 1.16(.64 – 2.12) | .53 | 2.01(.23-17.20) | .16 | 1.49(.85-2.65) | .50 | 2.25(.21-23.72) | .23 | .71(.41-1.24) | .99 | 18743215.7(.00-.) | .28 | (1.36(.78-2.35) |
| Parity | .91 | .96(.47-1.97) | .99 | .00(.00-.) | .28 | .69(.35-1.35) | .99 | .00(.00-.) | .11 | 1.73(.89-3.38) | .50 | 1.95(.28-13.68) | .15 | 1.66(.84-3.26) |
| Conception Type | .97 | .95(.09- 9.95) | .99 | .00(.00-.) | .66 | 1.69(.16-17.44) | .99 | .00(.00-.) | .64 | .61(.08-4.72) | .99 | .00(.00-.) | .54 | .48(.05-4.99) |
| Maternal Education | .26 | .54(.19-1.57) | .99 | .00(.00-.) | .43 | .69(.29-1.70) | .99 | .00(.00-.) | .84 | 1.09(.45-2.64) | .99 | 1763893.19(.00-.) | .44 | .1.41(.59-3.39) |
| Maternal Employment | .82 | .94(.53-1.66) | .25 | 3.03(.46-19.90) | .39 | 1.26(.74-2.15) | .99 | .00(.00-.) | .11 | 1.55(.91-2.65) | .99 | .00(.00-.) | .94 | .98(.58-1.66) |
| Paternal Education | .67 | .79(.26-2.35) | .99 | .00(.00-.) | .47 | .69(.26-1.86) | .99 | .00(.00-.) | .74 | 1.18(.45-3.11) | .99 | .00(.00-.) | .81 | .89(.33-2.35) |
| Paternal Employment | .51 | .67(.19-2.25) | .75 | 2.05(.02-178.37) | .11 | .44(.16-1.19) | .79 | 2.15(.01-584.93) | .49 | 1.40(.53-3.73) | .71 | .46(.01-26.63) | .99 | .00(.00-.) |
| Consanguinity | .98 | .99(.29-3.37) | .99 | .00(.00-.) | .96 | .97(.31-3.00) | .99 | .00(.00-.) | .98 | 1.02(.34-3.08) | .99 | 11(.00-.) | .29 | .55(.18-1.67) |
| Supplement Use (TYPE) | .78 | .85(.27-2.69) | .99 | .00(.00-.) | .25 | .54(.19-1.53) | .35 | .17(.00-6.86) | .24 | .54(.19-1.51) | .99 | .00(.00-.) | .19 | .1.99(.70-5.67) |
| Supplement Use (TIME) | .41 | .61(.19-2.01) | .99 | .00(.00-.) | .14 | 2.32(.76-7.12) | .99 | .00(.00-.) | .28 | .54(.18-1.64) | .35 | 4.83(.17-134.28) | .31 | .57(.19-1.69) |

Table 5: Biochemical biomarkers association with baseline sociodemographic characteristics using logistic regression. P-value <005 considered significant.

| PARAMETER | BIOCHEMICAL MARKER | | | | | | | | | | | |
| --- | --- | --- | --- | --- | --- | --- | --- | --- | --- | --- | --- | --- |
|  | Lead | | Calcium | | Copper | | Iron | | Magnesium | | HbA1c | |
|  | P. Value | Odds ratio (95 CI) | P. Value | Odds ratio (95 CI) | P. Value | Odds ratio (95 CI) | P. Value | Odds ratio (95 CI) | P. Value | Odds ratio (95 CI) | P. Value | Odds ratio (95 CI) |
| Maternal age | * | * | .12 | .33(.08-1.33) | .75 | 1.41(.17-11.54) | .86 | 1.06(.55-2.05) | .67 | 1.43(.28-7.31) | .05 | 3.76(1.02-13.84) |
| BMI | * | * | .75 | .79(.19-3.19) | .99 | .00(.00-.) | .97 | .99(.53-1.85) | .72 | .77(.19-3.16) | .71 | 1.25(.39-4.07) |
| Parity | * | * | .87 | 1.15(.22-6.08) | .99 | .00(.00-.) | .27 | .64(.29-1.42) | .99 | .00(.00-.) | .99 | .00(.00-.) |
| Conception Type | * | * | .08 | .07(.00-1.32) | .99 | .00(.00-.) | .89 | .85(.08-8.96) | .07 | .06(.00-1.24) | .99 | .00(.00-.) |
| Maternal Education | * | * | .99 | .00(.00-.) | .99 | 58420457.9(.00-.) | .56 | 1.37(.47-4.01) | .99 | .00(.00-.) | **.02** | 25.09(1.84-341.97) |
| Maternal Employment | * | * | .99 | .00(.00-.) | .99 | .00(.00-.) | .74 | 1.11(.61-2.02) | .99 | .00(.00-.) | .29 | .59(.22-1.58) |
| Paternal Education | * | * | .28 | .26(.02-2.96) | .45 | .29(.01-6.81) | .70 | 1.25(.39-3.97) | .89 | 1.20(.09-15.34) | **.04** | 16.73(1.14-245.36) |
| Paternal Employment | * | * | .69 | 2.18(.04-112.53) | .76 | 2.16(.02-289.63) | .59 | 1.39(.40-4.82) | .71 | 2.18(.04-126.14) | .69 | .49(.01-15.33) |
| Consanguinity | * | * | .99 | .00(.00-.) | .99 | .00(.00-.) | .17 | .37(.09-1.55) | .83 | 1.36(.08-23.01) | .99 | .00(.00-.) |
| Supplement Use (TYPE) | * | * | .99 | .00(.00-.) | .99 | .00(.00-.) | .05 | .28(.08-.97) | .99 | .00(.00-.) | .67 | .59(.06-6.42) |
| Supplement Use (TIME) | * | * | .38 | .22(.01-6.27) | .99 | .00(.00-.) | .52 | 1.51(.43-5.27) | .64 | 1.92(.12- 29.98) | .25 | 5.15(.32-83.17) |

Table 6: Biochemical biomarkers association with baseline sociodemographic characteristics using logistic regression. P-value <005 considered significant. * For Lead which association was not assessed.

| PARAMETER | DIETARY MACRONUTRIENTS | | | | | | | | | | |
| --- | --- | --- | --- | --- | --- | --- | --- | --- | --- | --- | --- |
|  | Protein | | CHO | | Fat | | Saturated fat | | Total Fiber | |  |
|  | P. Value | Odds ratio (95 CI) | P. Value | Odds ratio (95 CI) | P. Value | Odds ratio (95 CI) | P. Value | Odds ratio (95 CI) | P. Value | Odds ratio (95 CI) |  |
| Maternal age | .16 | 2.91(.65-13.11) | .94 | 1.03(.49-2.14) | .60 | 1.19(.63-2.24) | .36 | .76(.42-1.38) | .79 | 1.17(.38-3.63) |  |
| BMI | .23 | 1.92(.67-5.50) | .99 | 1.00(.48-2.09) | .63 | .85(.45-1.63) | .06 | .55(.29-1.02) | .30 | 2.02(.53-7.66) |  |
| Parity | .99 | .00(.00-.) | .16 | .51(.20-1.31) | .16 | 1.75(.79-3.85) | .88 | 1.05(.54-2.05) | .47 | .58(.14-2.51) |  |
| Conception Type | .99 | .00(.00-.) | .64 | 1.81(.15-22.64) | .99 | .00(.00-.) | .22 | .23(.02-2.40) | .99 | .00(.00-.) |  |
| Maternal Education | .48 | 1.84(.34-10.07) | .62 | 1.34(.42-4.26) | .45 | 1.49(.53-4.21) | .72 | .84(.33-2.16) | .81 | 1.26(.19-8.24) |  |
| Maternal Employment | .39 | 1.41(.64-3.09) | .08 | .56(.29-1.07) | .75 | 1.11(.59-2.09) | .09 | 1.64(.911-2.94) | .99 | .00(.00-.) |  |
| Paternal Education | .95 | .95(.19-4.75) | .68 | .76(.21-2.81) | .81 | 1.14(.38-3.47) | .70 | .82(.30-2.24) | .45 | .39(.04-4.21) |  |
| Paternal Employment | .48 | .67(.23-2.00) | .40 | .43(.061-3.06) | .81 | 1.12(.45-2.79) | .99 | .00(.00-.) | .62 | .45(.02-10.77) |  |
| Consanguinity | .94 | .93(.14-6.12) | .29 | .44(.09-1.99) | .70 | .78(.22-2.81) | .19 | 2.20(.68-7.10) | .38 | .33(.03-3.95) |  |
| Supplement Use (TYPE) | .25 | 3.75(.40-35.03) | .73 | 1.27(.33-4.83) | .89 | .92(.28-3.01) | .57 | 1.37(.47-3.98) | .63 | .56(.05-6.03) |  |
| Supplement Use (TIME) | .67 | .65(.09-4.55) | .09 | .28(.06-1.25) | .24 | 2.26(.56-9.14) | .28 | 1.91(.59-6.13) | .88 | .84(.08-8.48) |  |

Table 7: Macronutrients association with baseline sociodemographic characteristics using logistic regression. P-value <005 considered significant.

| PARAMETER | DIETARY MICRONUTRIENTS | | | | | | | | | | | |
| --- | --- | --- | --- | --- | --- | --- | --- | --- | --- | --- | --- | --- |
|  | Iron | | Folate | | Vit B12 | | Vit B1 | | Vit B2 | | Vit B3 | |
|  | P. Value | Odds ratio (95 CI) | P. Value | Odds ratio (95 CI) | P. Value | Odds ratio (95 CI) | P. Value | Odds ratio (95 CI) | P. Value | Odds ratio (95 CI) | P. Value | Odds ratio (95 CI) |
| Maternal age | .38 | 1.58(.57-4.37 | .97 | .98(.36-2.70) | .17 | 2.05(.73-5.78) | .58 | 1.23(.59-2.56) | .17 | 4.76(.50-45.06) | .94 | .97(.40-2.32) |
| BMI | .54 | .69(.22-2.22) | .87 | .92(.33-2.58) | .09 | .19(.03-1.37) | .31 | 1.52(.68-3.36) | .99 | .00(.00-.) | .98 | .99(.41-2.38) |
| Parity | .61 | .68(.16-2.96) | .17 | .23(.03-1.84) | .52 | .62(.14-2.69) | .71 | .84(.34-2.08) | .98 | 1.03(.09-10.71) | .16 | .43(.13-1.39) |
| Conception Type | .99 | .00(.00-.) | .99 | .00(.00-.) | .99 | .00(.00-.) | .68 | 1.67(.15-18.45) | .99 | .00(.00-.) | .47 | 2.47(.21-28.54) |
| Maternal Education | .24 | 3.31(.46-23.96) | 1.00 | 1.00(.11-8.42) | .47 | .43(.04-4.21) | .41 | 1.67(.49-5.64) | .51 | 2.79(.13-58.70) | .29 | .40(.08-2.14) |
| Maternal Employment | .99 | .00(.00-.) | .99 | .00(.00-.) | .99 | .00(.00-.) | .50 | .79(.41-1.56) | .99 | .00(.00-.) | .45 | 1.49(.53-4.22) |
| Paternal Education | .45 | .41(.04-4.21) | .99 | .00(.00-.) | .40 | .36(.03-3.87) | .93 | 1.06(.28-4.03) | .99 | .00(.00-.) | .22 | .34(.06-1.90) |
| Paternal Employment | .55 | .44(.03-6.45) | .66 | .58(.05-6.52) | .61 | .45(.02-10.06) | .40 | .41(.05-3.28) | .77 | .46(.00-84.69) | .47 | .43(.04-4.32) |
| Consanguinity | .99 | .00(.00-.) | .29 | 3.60(.32-40.23) | .72 | 1.58(.13-18.81) | .08 | 7.00(.78-62.53) | .99 | .00(.00-.) | .73 | .74(.13-4.12) |
| Supplement Use (TYPE) | .86 | .79(.07-9.71) | .99 | .00(.00-.) | .99 | .00(.00-.) | 1.00 | 1.00(.25-4.09) | .99 | .00(.00-.) | .14 | .19(.02-1.68) |
| Supplement Use (TIME) | .99 | .00(.00-.) | .79 | 1.40(.12-15.97) | .86 | .81(.08-8.19) | .33 | 2.33(.42-12.88) | .56 | 3.02(.04-395.18) | .54 | 1.77(.28-11.21) |

Table 8: Micronutrients association with baseline sociodemographic characteristics using logistic regression. P-value <005 considered significant.

| PARAMETER | DIETARY MICRONUTRIENTS | | | | | | | | | | | |
| --- | --- | --- | --- | --- | --- | --- | --- | --- | --- | --- | --- | --- |
|  | Vit B6 | | Biotin | | Choline | | Vit D | | Vit A | | Vit E | |
|  | P. Value | Odds ratio (95 CI) | P. Value | Odds ratio (95 CI) | P. Value | Odds ratio (95 CI) | P. Value | Odds ratio (95 CI) | P. Value | Odds ratio (95 CI) | P. Value | Odds ratio (95 CI) |
| Maternal age | .99 | 1.01(.39-2.56) | .22 | 4.23(.43-41.96) | .35 | 1.37(.71-2.62) | .14 | 2.18(.78-6.09) | .32 | .29(.03-3.25) | .91 | 1.04(.52-2.09) |
| BMI | .51 | .72(.27-1.94) | .99 | .00(.00-.) | .11 | 1.72(.88-3.34) | .09 | .20(.03-1.35) | .27 | 2.78(.45-17.19) | .55 | .81(.39-1.63) |
| Parity | .14 | .34(.085-1.39) | .89 | .84(.08-9.03) | .49 | 1.31(.61-2.82) | .58 | .65(.15-2.89) | .99 | .00(.00-.) | .48 | .74(.32-1.70) |
| Conception Type | .99 | .00(.00-.) | .99 | .00(.00-.) | .55 | .49(.05-5.16) | .99 | .00(.00-.) | .99 | .00(.00-.) | .99 | .00(.00-.) |
| Maternal Education | .96 | 1.04(.23-4.72) | .43 | 3.27(.17-61.24) | .68 | .79(.27-2.37) | .43 | .39(.04-4.12) | .84 | .78(.07-8.74) | .64 | 1.32(.42-4.19) |
| Maternal Employment | .99 | .00(.00-.) | .99 | .00(.00-.) | .16 | 1.69(.81-3.54) | .99 | .00(.00-.) | .63 | .75(.23-2.45) | .38 | .75(.39-1.42) |
| Paternal Education | .99 | .00(.00-.) | .99 | .00(.00-.) | .34 | .58(.19-1.77) | .39 | .36(.03-3.82) | .99 | .00(.00-.) | .77 | .84(.26-2.72) |
| Paternal Employment | .51 | .43(.04-5.25) | .94 | .79(.00-276.84) | .44 | .61(.17-2.19) | .61 | .45(.02-9.39) | .68 | .46(.01-19.29) | .99 | .00(.00-.) |
| Consanguinity | .29 | 3.46(.36-33.63) | .99 | .00(.00-.) | .08 | 3.30(.88-12.43) | .67 | 1.73(.15-20.58) | .85 | .76(.05-12.95) | .17 | .38(.09-1.54) |
| Supplement Use (TYPE) | .99 | .00(.00-.) | .99 | .00(.00-.) | .38 | 1.78(.49-6.37) | .99 | .00(.00-.) | .99 | .00(.00-.) | .45 | .58(.14-2.37) |
| Supplement Use (TIME) | .69 | 1.48(.21-10.37) | .59 | 3.33(.04-269.52) | .36 | .53(.13-2.06) | .81 | .73(.06-8.92) | .41 | 4.12(.14-122.23) | .76 | .80(.19-3.42) |

Table 9: Micronutrients association with baseline sociodemographic characteristics using logistic regression. P-value <005 considered significant.

| PARAMETER | DIETARY MICRONUTRIENTS | | | | | | | | | | | |
| --- | --- | --- | --- | --- | --- | --- | --- | --- | --- | --- | --- | --- |
|  | Vit K | | Vit C | | Zinc | | Calcium | | Phosphorus | | Magnesium | |
|  | P. Value | Odds ratio (95 CI) | P. Value | Odds ratio (95 CI) | P. Value | Odds ratio (95 CI) | P. Value | Odds ratio (95 CI) | P. Value | Odds ratio (95 CI) | P. Value | Odds ratio (95 CI) |
| Maternal age | .86 | .82(.09-7.79) | .92 | 1.04(.50-2.16) | .87 | .83(.09-7.86) | .84 | .79(.08-7.55) | .71 | 1.14(.57-2.27) | .27 | 1.99(.59-6.82) |
| BMI | .49 | .44(.04-4.68) | .39 | .71(.33-1.53) | .99 | .00(.00-.) | .43 | .43(.04-4.96) | .92 | 1.04(.51-2.12) | .79 | .82(.19-3.62) |
| Parity | .93 | 1.11(.10-12.13) | .39 | .68(.28-1.64) | .99 | .00(.00-.) | 1.00 | 1.00(.09-10.27) | .74 | .87(.38-1.99) | .44 | .46(.07-3.27) |
| Conception Type | .99 | .00(.00-.) | .76 | 1.46(.13-15.91) | .99 | .00(.00-.) | .99 | .00(.00-.) | .87 | 1.22(.11-13.21) | .99 | .00(.00-.) |
| Maternal Education | .49 | 3.22(.12-89.99) | .32 | 1.81(.56-5.89) | .50 | 2.80(.14-56.49) | .99 | .00(.00-.) | .61 | .74(.22-2.42) | .35 | 2.89(.32-26.42) |
| Maternal Employment | .99 | .00(.00-.) | .36 | 1.50(.63-3.58) | .99 | .00(.00-.) | .99 | .00(.00-.) | .42 | 1.38(.64-2.99) | .99 | .00(.00-.) |
| Paternal Education | .51 | 3.13(.10-94.12) | .81 | .86(.24-3.10) | .99 | .00(.00-.) | .49 | 3.38(.11-102.79) | .96 | .97(.28-3.31) | .99 | .00(.00-.) |
| Paternal Employment | .79 | .49(.00-100.91) | .38 | .40(.05-3.01) | .77 | .46(.00-79.74) | .78 | .47(.00-89.88) | .34 | .39(.06-2.70) | .68 | .46(.01-19.29) |
| Consanguinity | .99 | .00(.00-.) | .89 | .91(.21-3.93) | .99 | .00(.00-.) | .99 | .00(.00-.) | .64 | .71(.18-2.91) | .99 | .00(.00-.) |
| Supplement Use (TYPE) | .99 | .00(.00-.) | .34 | .49(.11-2.13) | .99 | .00(.00-.) | .37 | 5.42(.14-212.96) | .51 | .64(.17-2.45) | .99 | .00(.00-.) |
| Supplement Use (TIME) | .99 | .00(.00-.) | .70 | .75(.18-3.22) | .56 | 3.93(.04-374.14) | .57 | 3.92(.04-417.15) | .55 | 1.56(.36-6.78) | .41 | 4.12(.14-122.23) |

Table 10: Micronutrients association with baseline sociodemographic characteristics using logistic regression. P-value <005 considered significant.

| PARAMETER | DIETARY MICRONUTRIENTS | | | | | | | | | | | |
| --- | --- | --- | --- | --- | --- | --- | --- | --- | --- | --- | --- | --- |
|  | Copper | | Potassium | | Sodium | | Iodine | | Fluoride | | Chloride | |
|  | P. Value | Odds ratio (95 CI) | P. Value | Odds ratio (95 CI) | P. Value | Odds ratio (95 CI) | P. Value | Odds ratio (95 CI) | P. Value | Odds ratio (95 CI) | P. Value | Odds ratio (95 CI) |
| Maternal age | .46 | 1.35(.60-3.04) | .98 | 1.01(.39-2.59) | .23 | .62(.28-1.36) | .73 | .68(.08-6.20) | .59 | .68(.17-2.76) | .20 | 1.85(.72-4.70) |
| BMI | .35 | .64(.25-1.63) | .47 | .47(.26-1.87) | .45 | .76(.37-1.55) | .43 | .34(.02-4.94) | .36 | 1.89(.48-7.59) | .82 | 1.12(.43-2.86) |
| Parity | .31 | .57(.19-1.69) | .13 | .34(.08-1.36) | .08 | .42(.16-1.11) | .88 | .84(.08-8.72) | .53 | 1.63(.35-7.59) | .59 | 1.42(.39-5.15) |
| Conception Type | .48 | 2.40(.21-27.78) | .35 | 3.25(.27-39.05) | .99 | .00(.00-.) | .99 | .00(.00-.) | .58 | 2.17(.14-32.53) | .99 | .00(.00-.) |
| Maternal Education | .67 | .73(.17-3.15) | .45 | .52(.09-2.86) | .22 | 2.26(.62-8.22) | .99 | .00(.00-.) | .48 | .39(.03-5.21) | .49 | .50(.07-3.55) |
| Maternal Employment | .44 | 1.52(.53-4.29) | .99 | .00(.00-.) | .58 | 1.20(.63-2.29) | .99 | .00(.00-.) | .91 | 1.07(.34-3.34) | .31 | 1.76(.59-5.17) |
| Paternal Education | .23 | .35(.06-1.97) | .13 | .18(.02-1.66) | .59 | .67(.15-2.90) | .43 | 4.35(.12-161.24) | .89 | 1.20(.09-16.24) | .38 | 2.50(.32-19.53) |
| Paternal Employment | .53 | .45(.04-5.21) | .52 | .44(.04-5.39) | .68 | 1.49(.22-10.05) | .94 | .79(.00-253.65) | **.01** | .188 | 1.00 | .00(.00-.) |
| Consanguinity | .77 | .77(.14-4.32) | .90 | 1.13(.17-7.45) | .96 | 1.04(.23-4.70) | .99 | .00(.00-.) | .37 | 3.33(.25-45.11) | .38 | .40(.05-3.13) |
| Supplement Use (TYPE) | .47 | .52(.09-2.94) | .23 | .26(.03-2.39) | .86 | .89(.24-3.24) | .38 | 4.67(.15-143.20) | .37 | 3.33(.25-45.11) | .77 | .75(.11-5.24) |
| Supplement Use (TIME) | .82 | .82(.15-4.47) | .71 | 1.44(.20-10.23) | .72 | 1.28(.33-4.95) | .61 | 3.15(.04-277.46) | .99 | .00(.00-.) | .46 | 2.57(.21-31.71) |

Table 11: Micronutrients association with baseline sociodemographic characteristics using logistic regression. P-value <005 considered significant.

| PARAMETER | DIETARY MICRONUTRIENTS | | | |
| --- | --- | --- | --- | --- |
|  | Selenium | | Manganese | |
|  | P. Value | Odds ratio (95 CI) | P. Value | Odds ratio (95 CI) |
| Maternal age | .09 | 1.84(.92-3.67) | .06 | 2.21(.97-5.04) |
| BMI | .60 | 1.21(.59-2.49) | .77 | 1.14(.47-2.79) |
| Parity | 1.00 | 1.00(.44-2.29) | .34 | 1.71(.57-5.09) |
| Conception Type | .91 | 1.15(.11-12.43) | .99 | .00(.00-.) |
| Maternal Education | .11 | .30(.07-1.29) | .75 | 1.25(.32-4.94) |
| Maternal Employment | .56 | 1.26(.58-2.74) | .69 | .86(.40-1.84) |
| Paternal Education | .57 | .69(.19-2.45) | .81 | 1.20(.27-5.35) |
| Paternal Employment | .42 | .43(.05-3.41) | .99 | .00(.00-.) |
| Consanguinity | .74 | .79(.19-3.24) | .81 | .81(.14-4.54) |
| Supplement Use (TYPE) | **.03** | .09(.01-.79) | .43 | .49(.09-2.81) |
| Supplement Use (TIME) | .59 | 1.49(.34-6.53) | .50 | 1.86(.30-11.54) |

Table 12: Micronutrients association with baseline sociodemographic characteristics using logistic regression. P-value <005 considered significant.
